# Subclavian oblique-axis catheterization technique

**DOI:** 10.1186/s13054-017-1915-7

**Published:** 2017-12-27

**Authors:** Alessandro De Cassai, Helmut Galligioni

**Affiliations:** Emergency Department, Azienda Ospedaliera di Padova, Padua, Italy

We read with interest the work by Saugel et al. [1] about ultrasound-guided central venous catheter placement.

In their work the authors accurately describe “in-plane” and “out of plane” techniques for central venous catheter access. However, they describe only briefly the oblique-axis technique and for this approach only articles about internal venous jugular catheterization are cited [2, 3] and nothing in the literature about the oblique axis for subclavian access could be found.

**Fig. 1:**
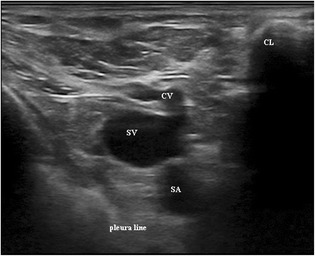
Starting point of subclavian vein. SV subclavian vein, SA subclavian artery, CV cephalic vein, CL clavicle

**Fig. 2:**
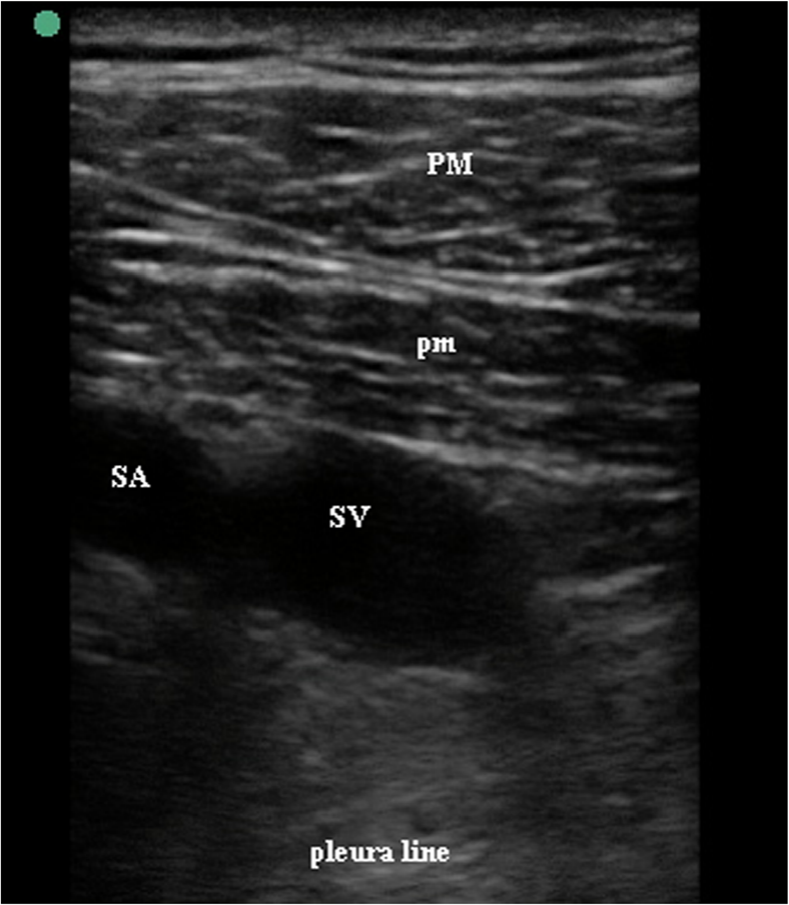
Oblique-axis view of subclavian vein and artery. SV subclavian vein, SA subclavian artery, PM pectoralis major, pm pectoralis minor

In our experience we make regular use of the oblique-axis technique for placing subclavian catheters with a high success rate and a low rate of complications. An important aspect for a successful procedure is patient positioning; in fact, the best visualization of the subclavian vein and artery is, in our experience, possible with a 90° abduction of the arm. After aseptic preparation the ultrasound probe is covered with a protective plastic sheath. Ultrasound inspection is started laterally near the axilla to identify the axillary vein and artery in a classic “out of plane” view, after which the vessels are followed until the joint point of the axillary vein with the cephalic vein (Fig. 1): this is the anatomic subclavian starting point. After a 45° rotation of the probe an oblique view is then possible (Fig. 2). It is an easy method with great advantages in comparison with “in-plane” and “out of plane” views because it permits both visualization of the tip of the needle and important anatomical structures such as the artery or vein (both with an oval shape due to projection) and, moreover, sliding of the lung.
